# Molecular Characterization of Vitellogenin and Its Receptor Genes from Citrus Red Mite, *Panonychus citri* (McGregor)

**DOI:** 10.3390/ijms16034759

**Published:** 2015-03-02

**Authors:** Rui Zhong, Tian-Bo Ding, Jin-Zhi Niu, Wen-Kai Xia, Chong-Yu Liao, Wei Dou, Jin-Jun Wang

**Affiliations:** Key Laboratory of Entomology and Pest Control Engineering, College of Plant Protection, Southwest University, Chongqing 400715, China; E-Mails: zhongrui19890326@gmail.com (R.Z.); tianboding@gmail.com (T.-B.D.); jinzhi.niu@ugent.be (J.-Z.N.); wenkaixia0409@gmail.com (W.-K.X.); leochongyu@gmail.com (C.-Y.L.); anwdou@gmail.com (W.D.)

**Keywords:** *Panonychus citri*, vitellogenin, vitellogenin receptor, cloning, relative expression

## Abstract

The production and uptake of yolk protein play an important role in the reproduction of all oviparous organisms. Vitellogenin (Vg) is the precursor of vitellin (Vn), which is the major egg storage protein, and vitellogenin receptor (VgR) is a necessary protein for the uptake of Vg into developing oocytes. In this paper, we characterize the full-length Vg and VgR, *PcVg1* and *PcVgR*, respectively, of the citrus red mite *Panonychus citri* (McGregor). The *PcVg1* cDNA is 5748 nucleotides (nt) with a 5553-nt open reading frame (ORF) coding for 1851 amino acids (aa), and the *PcVgR* is 6090 nt, containing an intact ORF of 5673 nt coding an expected protein of 1891 aa. The *PcVg1* aa sequence shows a typical GLCG domain and several K/RXXR cleavage sites, and *PcVgR* comprises two ligand-binding domains, two epidermal growth factor (EGF)-like regions containing YWTD motifs, a transmembrane domain, and a cytoplasmic domain. An analysis of the aa sequences and phylogenetics implied that both genes were genetically distinct from those of ticks and insects. The transcriptional profiles determined by real-time quantitative PCR in different developmental stages showed that both genes present the same expressional tendencies in eggs, larvae, nymphs, and adults. This suggested that the biosynthesis and uptake of *PcVg* occurs coordinately. The strong reproductive capacity of *P. citri* has been hypothesized as an important factor in its resistance; consequently, understanding the molecular mechanisms regulating Vg and VgR are fundamental for mite control.

## 1. Introduction

The citrus red mite, *Panonychus citri* (McGregor) (Acari: Tetranychidae) is a worldwide agricultural pest that devastates fruit trees [1,2]. To control this mite, intensive application of acaricides has been performed in the fields, which has led to strong resistance to various acaricides, challenging the sustainable control of this pest [3]. At present, it is a destructive pest in citrus orchards mainly because of its ability to evolve resistance to many classes of insecticides. There is an urgent need to develop new pest control strategies by targeting vital genes. Vitellogenin (Vg) is the precursor of vitellin (Vn), which provides amino acids (aa), lipids, carbohydrates, vitamins and other nutrients for the development of the embryo. Additionally, it may function in the control of egg buoyancy and immune responses of fish [4,5]. The synthesis of Vg is regulated by different hormones. In many insects it is induced by juvenile hormone, whereas in ticks it is induced by ecdysteroids [6]. In arthropods, Vg is usually synthesized in the fat body, then secreted into the hemolymph and taken up by the vitellogenin receptor (VgR) located in clathrin-coated pits on the external surface of growth-competent oocytes. Vg biosynthesis and uptake play important roles in mite reproduction; therefore, members of these pathways could be promising for new acaricide development [7].

The number of Vg genes varies in different arthropod species [8]; however, there is a large (200–700 kDa) homologous phosphoglycolipoprotein present in all oviparous species. The transcripts of *Vg* genes are 5–8 kb in length and belong to the large lipid transfer gene family. Vg share similar structural motifs, such as an *N*-terminal lipid binding domain (LPD_N), the unknown functional region (DUF1943), a von Willebrand factor type D similar domain (vWD), cleavage sites (R/KXXR) and *C*-terminal GLCG domain [9]. The fat body is not the only vitellogenic tissue; the Cyclorapha ovarian follicular epithelium produces yolk protein (YP) [10]. For insect at least, Vg is no longer considered a female-specific protein, as the expression and synthesis of Vg in larvae and males of *Apis mellifera* have been reported [11]. Vg uptake is a dramatic example of a receptor-mediated endocytosis pathway in many species [12]. VgR is an ovary-specific member, belonging to the low-density lipoprotein receptor (LDLR) superfamily [13]. LDLR family members have five distinct domains: ligand-binding domains (LBD), epidermal growth factor (EGF)-like repeats, β-propeller domains (YWXD motif), a transmembrane domain anchoring the receptor to the plasma membrane, and a cytoplasmic domain [14]. In insects, the *VgR* gene encodes a protein of 180–214 kDa, which is approximately twice as large as that in vertebrates (95–115 kDa) [15].

To date, Vg and VgR genes have been isolated from many species of both vertebrates, such as *Morone saxatilis* and *Oncorhynchus mykiss* [16,17,18], and invertebrates, such as *Aedes aegypti* [19], *Solenopsis invicta* [20], *Blattela germanica* [21], *Leucophaea maderae* [22], the crustacean *Scylla serrata* [23] and the nematode *Caenorhabditis elegans* [24]. In *Acarina*, the full-length transcript sequences of Vg and VgR were sequenced from two species of tick, *Dermacentor variabilis* [9,25,26] and *Haemaphysalis longicornis* [12,27], as well as two species of mite *Varroa destructor* [28] and *Tetranychus urticae* [29]. The amino acid sequence of Vg2 in *D. variabilis* has a typical GLCG domain and several TOM cleavage sites, which is present in most isolated Vgs [9]. Each of VdVg1 and VdVg2 proteins has greater similarity with Vg1 and Vg2 proteins from ticks, respectively, than between themselves [28]. However, the molecular information and roles of Vg and VgR during the growth and reproduction of the important pest species *P. citri* are still unclear.

Therefore, in this study, we report: (1) the complete aa sequence of the *P. citri* Vg gene, *PcVg1* (GenBank Accession number: KC978893), and the VgR gene, *PcVgR* (GenBank Accession number: KC978894); (2) their molecular characteristics and structural comparisons with related genes from other species; and (3) the relative expression levels of *PcVg1* and *PcVgR* during different *P. citri* developmental stages. These results will improve our understanding of Vg biosynthesis and its role in mite development, and help further research on new *P. citri* control strategies based on disrupting Vg biosynthesis.

## 2. Results

### 2.1. Nucleotide (nt) and Deduced Amino Acids (aa) Sequences

The complete cDNA of *PcVg1* contained an ORF of 5553 nt, encoding 1851 aa. The cDNA sequence included the start codon ATG at position 62–64 and the stop codon TAA at position 5615–5617. The 5'-untranslated region (UTR) was 61 bp, and the 3'-UTR was 131 bp. The theoretical molecular weight was 210.57 kDa, and pI was 6.78. The putative signal peptide, consisting of 17 aa, MKIALFVLGLFVVSAFA, was located at the *N*-terminus of the deduced aa sequence, with a cleavage site between aa 17 and 18. The conserved domains of the *PcVg1* aa sequence are an LPD_N (27–754 aa), a DUF1943 domain (787–1057 aa) and a vWD (1521–1671 aa). *PcVg1* presented the typical *Vg* characteristics, including the GLCG domain and R/KXXR cleavage sites (Figures S1).

The full-length cDNA of *PcVgR* was 6090 nt and contained an ORF of 5673 nt, encoding 1891 aa. The start codon ATG was located at position 231–233, and the stop codon TAA was at position 5904–5906. The 5'-UTR was 230 bp, and the 3'-UTR was 184 bp. The theoretical molecular weight was 211.46 kDa, and the pI was 5.45. The signal peptide consisted of a sequence of 25 aa, MWPKLVGYSISFSLLFVISFIRVEG. The predicted protein sequence of *PcVgR* revealed the typical LDLR family receptor features. *PcVgR* exhibited two LBDs with four class A cysteine-rich repeats in the first domain and eight repeats in the second domain. Each repeat contained six cysteine residues. There were eight EGF-like repeats, with six cysteines each. The YWTD motif was present in three groups of six between the repeats [30]. A transmembrane domain (TMD) was found at aa residues 1768–1790, and a cytoplasmic domain was predicted at aa residues 1791–1891. In addition, potential internalization signals were noted at position 1873–1876 (Supplementary Figure S2).

### 2.2. Phylogenetic Analyses of PcVg1 and PcVgR

**Figure 1:**
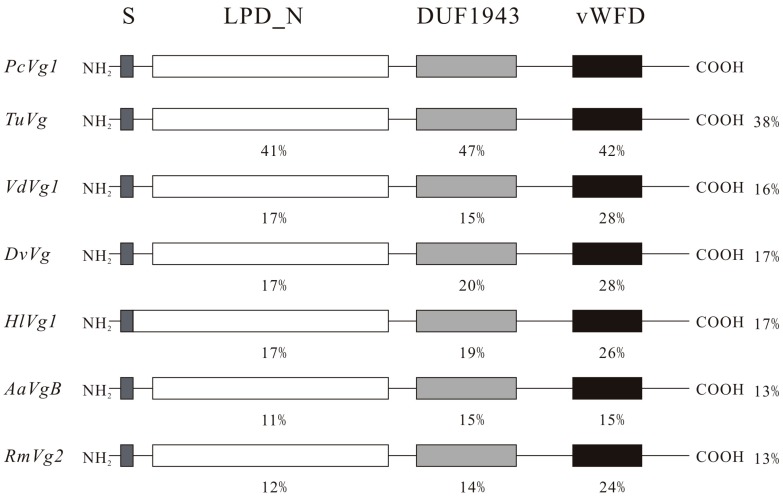
Schematic comparison of the primary protein structures of *Panonychus citri* vitellogenin 1 (*PcVg1*) with those of other arthropod species. The percentage identity compared to *PcVg1* for the specific domain is shown. The ratio (%) on the right side indicates the overall identity as compared with the *PcVg1*. S, signal peptide; LPD_N, *N*-terminal lipoprotein domain; DUF1943, a domain of unknown function; vWD, von Willerand Factor type D domain. Sequences were taken from the *Tetranychus urticae* genome and GenBank database (accession ID or No. in parentheses): *TuVg*, *T. urticae* (tetur27g01310); *VdVg1*, *Varroa destructor* (JQ974976); *DvVg*, *Dermacentor variabilis* (AY885250); *HlVg1*, *Haemaphysalis longicornis* (AB359899); *AaVgB*, *Aedes aegypti* (AAQ92367); and *RmVg2*, *Rhyparobia maderae* (BAD72597).

The amino acid sequence of *PcVg1* had three domains: LPD_N, DUF1943 and vWD, which were in similar locations relative to other animal species. A BLAST analysis revealed that *PcVg1* shared a 13%–17% overall aa identity to those of ticks and insects (Figure 1). The phylogenetic analysis of the aa sequence similarities between the *PcVg1* and 12 other *Vg* genes from other species was conducted. The genes from Acari segregated into a single clade, separate from the clades of genes from Insecta and Crustracea. *PcVg1* was most closely related to *TuVg* of *T. urticae* (Figure 3A).

**Figure 2:**
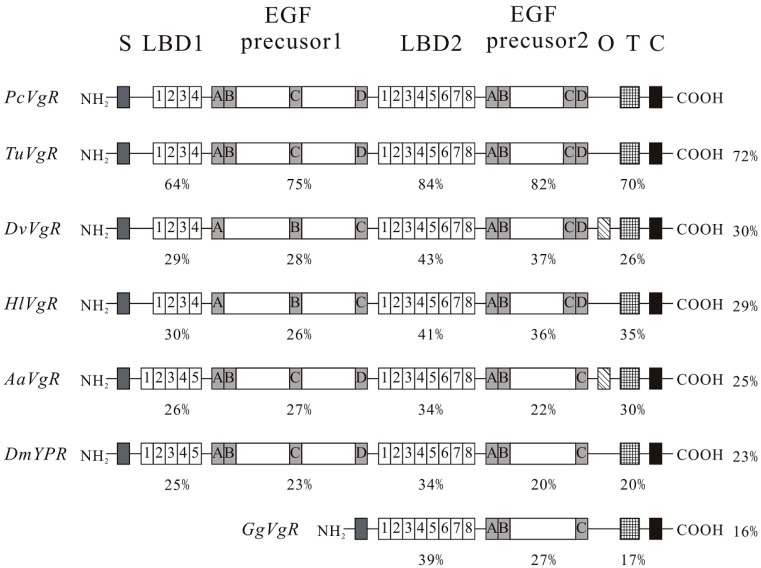
Schematic comparison of the primary protein structures of *Panonychus citri* vitellogenin receptor (*PcVgR*) with those of other arthropod species. The cysteine-rich repeats in the ligand binding domains (LBDs) are shown with numbers 1–8. The cysteine-rich repeats in the epidermal growth factor (EGF)-precursor domains are indicated with letters A–D. The percentage identity compared with *PcVgR* in a specific domain is shown. The ratio (%) on the right side indicates the overall identity as compared with *PcVgR*. S, signal peptide; O, *O*-linked sugar domain; T, transmembrane region; C, cytoplasmic tail. Sequences were taken from the *Tetranychus urticae* genome and GenBank database (accession ID or No. in parentheses): *TuVgR*, *T. urticae* (tetur27g01310); *DvVgR*, *Dermacentor variabilis* (DQ103506); *HlVgR*, *Haemaphysalis longicornis* (AB299015); *AaVgR*, *Aedes aegypti* (L77800); *DmYPR*, *Drosophila melanogaster* (DMU13637); and *GgVgR*, *Gallus gallus* (X80207).

**Figure 3:**
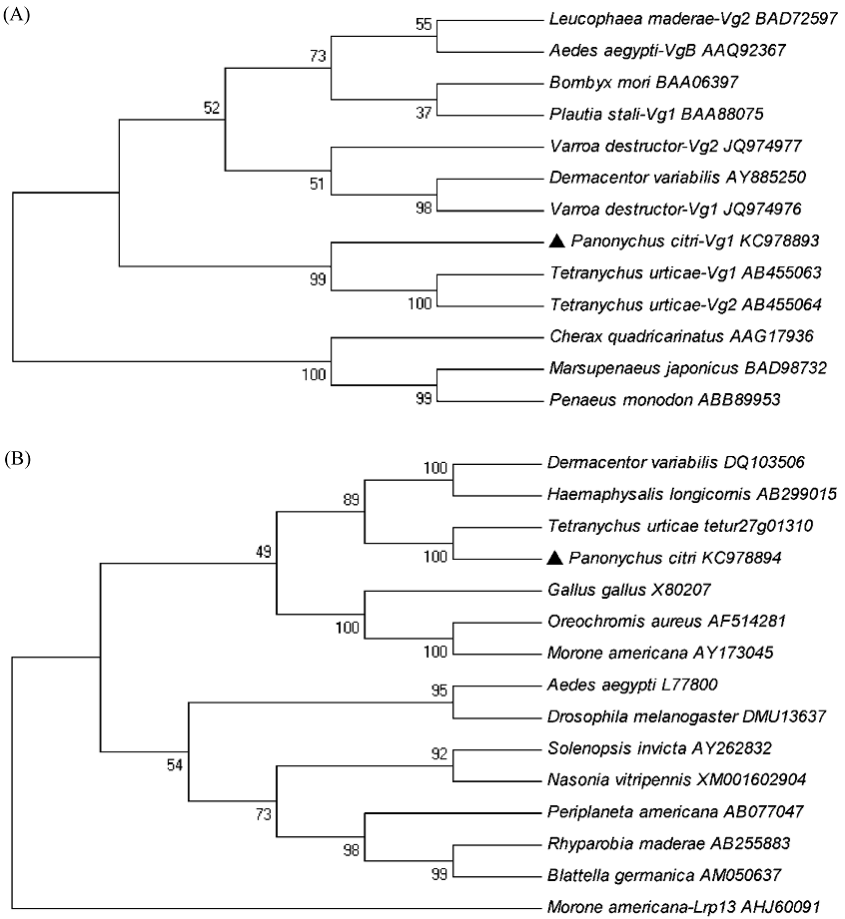
Phylogenetic relationship of vitellogenin 1 (*PcVg1*, ▲) (**A**) and vitellogenin receptor (*PcVgR*, ▲) (**B**) of *Panonychus citri* with those of other arthropod species. The phylogenetic tree was inferred using the neighbor-joining method. Numbers above the branches are bootstrap support values. The labels are organism names and GenBank accession numbers or Gene IDs.

Comparing the *PcVgR* with other species’ *VgR*s in NCBI revealed a high degree of structural similarity. The modular arrangement of the amino acid sequence motifs (LBDs and EGF-like domains) in six animal receptors was typical, but there were some differences among species. *PcVgR* showed 72% overall aa identity to *TuVgR*, but 30% and 29% when compared with *DvVgR* and *HlVgR*, respectively. Additionally, the overall aa identity was 25% to *AaVgR* and 23% to *DmYPR* (*Drosophila melanogaster*) (Figure 2). The phylogenetic tree showed that both of Vg and VgR in Acarina formed a separate group, and that they had a closer ancestry with fish than insects. *PcVgR* and *TuVgR* formed a single clade, indicating that they have the closest ancestry (Figure 3B).

### 2.3. Developmental Expression Profiles

**Figure 4:**
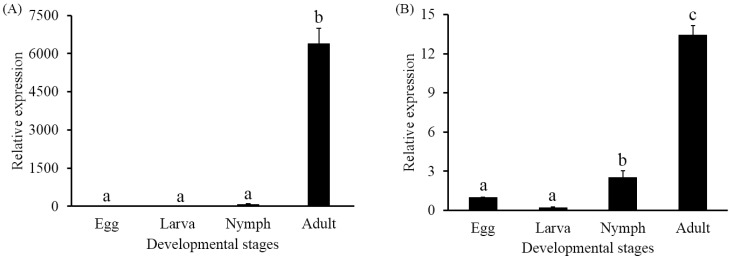
Expression profiles analysis of vitellogenin 1 (*PcVg1*) (**A**) and vitellogenin receptor (*PcVgR*) (**B**) of *Panonychus citri* at different developmental stages. The results are shown as the mean ± SE (*n* = 3). Different letters on the error bars indicate significant differences among the developmental stages. Significant differences between four developmental stages were assayed by one-way analysis of variance (ANOVA) with a *p*-value < 0.05.

The transcriptional expression levels of *PcVg1* and *PcVgR* were detected in all life stages (egg, larva, nymph, and female adult). For both genes, the expression levels in the adult stage were significantly higher than in the other developmental stages. The mRNA expression levels of *PcVg1* in adult and nymph were 6396.94- and 90.27-fold higher, respectively, than in the egg, while in the larva it was 0.48-fold lower (Figure 4A). The mRNA expression levels of *PcVgR* were 13.45- and 2.51-fold higher in adult and nymph, respectively, than in the egg, while it was 0.20-fold lower in the larva (Figure 4B).

## 3. Discussion

In this study, the complete cDNA sequences of *PcVg1* and *PcVgR* were obtained. Similar to those of insects, *Acari Vg* genes are 5–6 kb in length [8,25,26]. The GL/ICG motif is considered the most highly conserved Vg domain and is essential for the oligomerization of the vertebrate Vn. The motif has been mutated in different species of *Acarina*, such as *P. citri*, *T. urticae* and the soft tick *Ornithodoros moubata*, which contain the GLCG motif [31,32], the hard tick *D. variabilis*, which contains GLCS residues [9], and the parasitic mite *V. destructor*, which contains GVCG residues. In most holometabolous insects, the *Vg* gene product is cleaved by subtilisin-like endoproteases at a consensus cleavage site, K/RXXR, and the gene’s transcription product is cleaved into two. In hemimetabolous insects, multiple cleavage sites are found, resulting in the Vg being processed into several different molecular weights [33].

The *PcVgR* is comprised of several modular elements that are highly conserved in the LDLRs family. There were six cysteine residues in each ligand-binding domain involved in forming the three disulfide bridges (C1–C3, C2–C5, and C4–C6) [7]. In this study, we found that the *PcVgR* had two clusters of ligand binding repeats, with four modules in the first cluster and eight in the second cluster, which was consistent with *SiVgR* from the fire ant *Solenopsis invicta* [20]. Following the LBD is the EGF-precursor homology domain, which includes EGF-like repeats and YWXD repeats. Like the LBD, EGF-like repeats have six cysteine residues, but the arrangement is different (C1–C3, C2–C4, and C5–C6). Here, eight EGF-like repeats were found in *PcVgR*, four in each cluster, which is one more repeat than in the tick EGF precursor 1 and one more repeat than in the insect EGF precursor 2 (Figure 2). There are six YWTD repeats in each group, and they fold into a compact structure known as a six-bladed β-propeller domain [33]. The *O*-linked sugar domain (OLSD) is a short region, rich in serine and threonine residues [30]. Similar to the *HlVgR*, the *PcVgR* did not contain an OLSD, which is different from the *AaVgR* and *DvVgR*, indicating that the presence of this domain is not universal among invertebrate VgRs [18]. In vertebrate VgRs, the absence of OLSD has also been reported [34,35]. There is at least one NPXY motif in the cytoplasmic domains of all LDLRs [36]. The insect VgR contains a NPXY internalization signal in addition to the LI/LL found in most other LDLR family receptors [19,20,21], suggesting that the insect Vg/YPRs are stricter in harboring LI/LL signals than NPXY signals [13]. The coexistence of LI/LL and NPXY generally occurs in ticks, but there is no LI/LL in the aa sequence of *PcVgR* and *TuVgR*.

Among the Tetranychidae, the *VgR* structures of *P. citri* and *T. urticae* are highly similar, and they showed a 72% overall aa identity, while that of the *PcVg1* genes was 38%. Both *PcVg1* and *PcVgR* had low overall identities when compared with those of insects. The similarity was even lower when compared with those of other vertebrates. In general, the structural similarity of *PcVgR* was higher than that of *PcVg1*. From the published sequences, we proposed that the VgR, as a single gene transcript, is more conserved than the Vg, which is encoded by multiple genes. Thirteen *Vg* and *VgR* aa sequences of different species were selected to construct two separate phylogenetic trees. They were divided into three groups: Insect, *Arachnida*, and *Malacostraca*. Both the *Vg* and *VgR* of *P. citri* and *T. urticae* were sorted into separate clades, indicating their close ancestry. Interestingly, the *Vg* of the parasitic mite, *V. destructor*, was separated from the species of Tetranychidae, and formed a single clade with the ticks. When the *Vg* and *VgR* aa sequences of *P. citri* were compared with others, they had high similarities with those of *T. urticae*, but had low overall identities with *V. destructor* and ticks. Recent molecular studies revealed that the deduced aa sequences of the four *TuVg* cDNA fragments were distinct from those of ticks [32].

To characterize the developmentally specific expression profiles of *PcVg1* and *PcVgR*, we performed qPCR analyses using total RNA extracted from four developmental stages. The results showed that both gene transcripts were expressed in all of the tested stages and shared the same trend. This implied that the synthesis and uptake of Vg occurred coordinately. A previous report indicated that HlVgR transcripts were detected in eggs, larvae, nymphs and unfed female ticks by RT-PCR, and it was expressed at a higher level in eggs than in unfed female ticks [12]. In this study, however, the *PcVgR* expression in fed adult females was shown to be almost 12-fold higher than in eggs. In *Lepidopteran* insect, *Spodoptera litura*, VgR mRNA was first transcribed in sixth day female pupae and the maximum level of VgR mRNA appeared in 36 h-old adults [37]. Similarly, the *LmVgR* mRNA existed throughout the ovrian development and the transcriptional level was especially high in the previtellogenic periods [22]. Whereas the mRNA level of VgR in the mosquito, *A. aegypti*, increased rapidly after adult emergence and continued to increase during the vitellogenic periods [38]. Therefore, further work is needed to confirm whether a food shortage has an impact on *PcVgR* expression.

## 4. Experimental Section

### 4.1. Mites

The *P. citri* colony was collected from the citrus nursery at the Citrus Research Institute, Chinese Academy of Agricultural Sciences, Chongqing, China. The mites were kept on citrus seedlings in incubators at 25 ± 1 °C, 75%–80% relative humidity (RH) with 14:10 light:dark (L:D) photoperiod for several generations. To collect the different stages of the mites (egg, larva, nymph and adult), circular lemon leaf discs were prepared (25-mm diameter) from leaves collected in a pesticide-free orchard and washed by ddH_2_O. The leaf discs were placed on sponges in water-saturated Petri dishes (9-cm diameter). Twenty adult females were transferred to one leaf disc to lay eggs for 24 h before being removed. Molting was used as the criterion to indicate the next developmental stage [39]. The mite samples (1000 eggs, 800 larvae, 600 nymphs and 300 female adults) were collected into centrifuge tubes, separately, and stored at −80 °C for further experiments. Four biological repeats of each experiment were run for each developmental stage.

### 4.2. RNA Extraction and Transcription

Total RNA used for cloning was extracted from 200 female adults of *P. citri* using the RNeasy Plus Micro Kit (Qiagen GmbH, Hilden, Germany), according to the manufacturer’s instructions. The RNA sample was dissolved in 20 μL diethylpyrocarbonate (DEPC)-treated H_2_O and assessed at an absorbance ratio of OD 260/280 (1.8–2.1) using a NanoVue spectrophotometer (GE Healthcare, Fairfield, CT, USA). The RNA integrity was confirmed using 1% agarose gel electrophoresis. The first-strand cDNA was synthesized using a SMARTer™ RACE cDNA Amplification Kit (Clontech, Mountain View, CA, USA) for cloning and a PrimeScript^®^ RT reagent Kit (Takara, Dalian, China) for real-time quantitative PCR (qPCR).

### 4.3. Cloning Full-Length PcVg1 and PcVgR cDNAs

One *Vg* and six *VgR* cDNA fragments were identified in the transcriptome of *P. citri* [40]. Primers were designed based on those for the coding-region length (5547 bp) of the *VgR* cDNA of *T. urticae* (Table 1) and were positioned on the three fragments of *P. citri* to join the segments into one sequence. Two specific primers were designed for 5' and 3' rapid amplification of cDNA ends (RACE) for each gene (Table 1). The PCR program was as follows: an initial denaturation at 94 °C for 3 min, followed by 35 cycles of amplification at 94 °C for 30 s, 52–59 °C (depending on the annealing temperatures of the primers) for 30 s, 72 °C for 30–60 s (based on the predicted length of the amplified products), and a final extension at 72 °C for 10 min. The PCR products were separated in 1% agarose gels, and the bands were purified using a Gel Extraction Mini Kit (Watson Biotechnologies, Inc., Shanghai, China). Then, the fragments were cloned into the pGEM-T easy vector (Promega, Fitchburg, MA, USA), and the plasmid clones were sequenced at the Beijing Genomics Institute, Beijing, China.

**Table 1:** Primers used for cloning.

Experiments	Primer Names and Sequences (5' to 3')	Product Length (bp)
3'-RACE	Vg1-S1: GGTCATTCCGAACTCTTTCC	1939
	Vg1-S2: AGACGGTACCCACTACAACG	1941
	VgR-S1: ACATTTAGAGCCATTCGG	454
	VgR-S2: CCAGTGAGTTTGGACGAT	303
5'-RACE	Vg1-A1: TTCAGCGAGAACCATTTGGA	1552
	Vg1-A2: TGAGTGTTCGGTGTTGGTGA	1468
	VgR-A1: ATCGAGGTGATTCATCGTCA	1089
	VgR-A2: TGAGCCATCGAAACAATCCT	938
Splicing primer	VgR-S-S1: ACCCTGAGAAAGGTCTTATG	
	VgR-S-A1: CAATGAAGGAGCACAATGT	1481
	VgR-S-S2: GTGCAATTACCTGTCCAC	
	VgR-S-A2: CAACCCAATAAATCATTTTC	1197
Oligo (dT) primer	UPM:CTAATACGACTCACTATAGGGCAAGCAGTGGTATCAACGCAGAGT	
	NUP: AAGCAGTGGTATCAACGCAGAGT	
Full-length confirmation	Vg1-F-S1: TCGAACATGAAGATCGCTC	
	Vg1-F-A1: GTGAGTTCCTTAAGAGCCAAGT	1952
	Vg1-F-S2: TCAGACTCACAAATCGATTACC	
	Vg1-F-A2: TCTTCGTGGGCAAGAGTT	2267
	Vg1-F-S3: AATACATCGCTAACCTTACCTG	
	Vg1-F-A3: GAGATGATTTAAATGCCTCG	1682
	VgR-F-S1: CTCAAAATGTGGCCTAAACTAGTC	
	VgR-F-A1: TCGGGGATAAAACTGGATG	1909
	VgR-F-S2: TCTCGTCGTGGTCGTTCA	
	VgR-F-A2: CCTCGTCCTCCTGGTTAACAC	1873
	VgR-F-S3: GTTGCTCTGATGGTCATTGT	
	VgR-F-A3: TTAAACTTTTATAAAAACACGTTGG	2096

The DNAMAN software (DNAMAN 6.0.3, Lynnon BioSoft, Quebec, QC, Canada) was used to assemble the cloned genes’ cDNA fragments to putative full-length *PcVg1* and *PcVgR* sequences. Then, primer pairs were designed to amplify the open reading frames (ORF). Three specific primer pairs (Table 1) were used to confirm the ORF of each gene.

### 4.4. Sequence and Phylogenetic Analyses

The sequence similarities were analyzed using BlastP programs in the NCBI databases (Available online: http://www.ncbi.nlm.nih.gov/), and the conserved domains were identified using the ExPASy Prosite SCAN (Available online: http://www.expasy.org/tools/) and SMART (Available online: http://smart.embl-heidelberg.de/). Both gene sequences were edited with DNAMAN. The molecular weight and isoelectric point (pI) of the deduced protein sequences were predicted using the ExPASy Molecular Biology Server of the Swiss Institute of Bioinformatics (Available online: http://web.expasy.org/compute_pi/). The signal peptide was predicted using the SignalP 4.1 Server (Available online: http://www.cbs.dtu.dk/services/SignalP/). The transmembrane region was analyzed using the TMHMM Server (v.2.0) (Available online: http://www.cbs.dtu.dk/services/TMHMM/). MEGA5 [41] was used for constructing the phylogenetic tree using the neighbor-joining method. Bootstrap values were calculated on 1000 replications.

### 4.5. qPCR

The primers for the qPCR of both genes were designed using Primer 3 (v.0.4.0) (http://bioinfo.ut.ee/primer3-0.4.0/) (Table 2), and cDNA samples were diluted to 1/3, 1/9, 1/27, 1/81, and 1/243 to calculate the efficiency of amplification. *GAPDH* was used as a stable reference gene based on a previous evaluation [42]. The relative mRNA expression levels of the two genes in egg, larva, nymph and female adult were determined. The qPCR was performed using a Stratagene Mx3000P thermal cycler (Agilent Technologies, Inc., Wilmington, NC, USA). The reaction volume was 20 μL, containing 1 μL template cDNA, 10 μL SYBR *Premix Ex*Taq™ II (Perfect Real Time) (Takara), 1 μL each primer and 7 μL double distilled water. The reaction protocol was: 95 °C for 30 s, followed by 40 cycles of 95 °C for 5 s and 60 °C for 30 s. At the end, a melting cycle (from 60 to 95 °C) was included. The relative expression level of *PcVg1* and *PcVgR* at four developmental stages was calculated according to the 2^−ΔΔ*C*t^ method [43].

**Table 2:** Primers used for qPCR.

Genes	GenBank No.	Primer Names and Sequences (5' to 3')	Amplicon Sizes (bp)	Amplification Efficiency (%)
*PcVg1*	KC978893	Vg1-RTS: GCCTCAAACGAAGCTCAATC	183	97.7
		Vg1-RTA: AGCCAAAGCGTCGAGTAAAA		
*PcVgR*	KC978894	VgR-RTS: TTGTTTCGATGGCTCAGATG	150	108.9
		VgR-RTA: TCACCGTGTGGACAATCAGT		
*GAPDH*	HM582445	GAP-RTS: CTTTGGCCAAGGTCATCAAT	159	108.1
		GAP-RTA: CGGTAGCGGCAGGTATAATG		

### 4.6. Statistical Analysis

The differences for *PcVg1* and *PcVgR* among four developmental stages were analyzed by one-way analysis of variance (ANOVA) with a *p*-value less than 0.05 and means were separated with least significant difference (LSD) method using SPSS (v.16.0, SPSS Inc., Chicago, IL, USA).

## 5. Conclusions

In summary, we identified and characterized *PcVg1* and *PcVgR* cDNAs in *P. citri.* Both genes were highly conserved in their primary structure when compared with those of other species. The expression profiles of both genes in the four developmental stages demonstrated the expression of *PcVg1* and *PcVgR* was coordinated and shared similar trends. This is the first report describing complete sequences and expression profiles of Vg and VgR from *P. citri*. In the future, we will focus on the other *Vg* genes in *P. citri*, and the action mechanisms of *PcVg* and *PcVgR* in the pathway.

## Supplementary Materials

Supplementary materials can be found at http://www.mdpi.com/1422-0067/16/03/4759/s1.
