# Supplementary material for: Molecular Characterization of Vitellogenin and Its Receptor Genes from Citrus Red Mite, Panonychus citri (McGregor)
Source: Int J Mol Sci. 2015 Mar 2;16(3):4759–73. doi: 10.3390/ijms16034759 (PMC4394447; doi:10.3390/ijms16034759)
Supplement: Supplementary file 1 [file ijms-16-04759-s001.pdf]

Supplementary Information

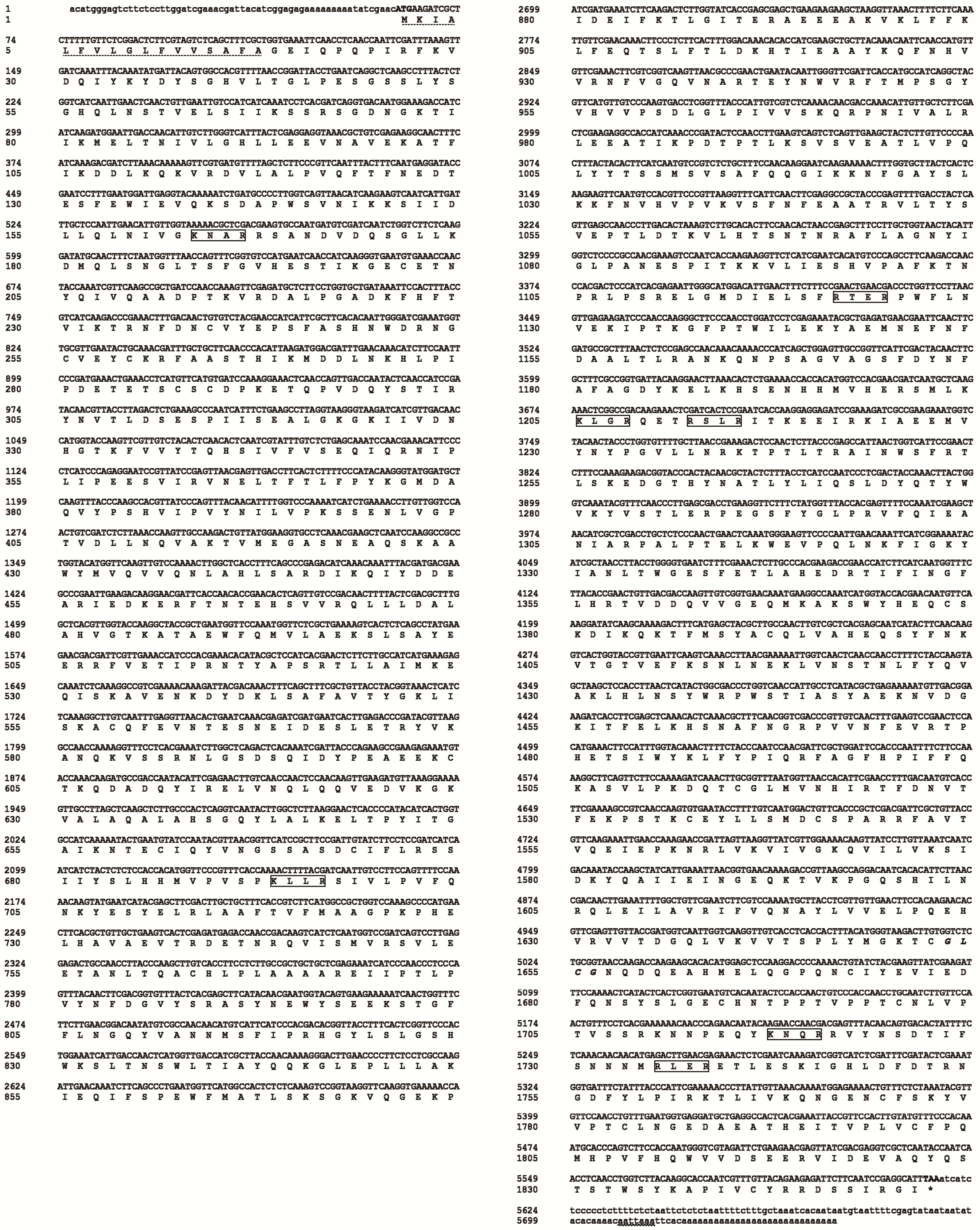

Figure S1. Nucleotide and amino acid sequences of vitellogenin 1 (Vg1) from *Panonychus citri* (*PcVg1*). The ATG start codon and TAA stop codon are in bold, and the signal peptide is underlinedwith a dashed line. The cleavage signal K/RXXR is indicated by an open box. The GLCG conserved motif is bold and italicized. The polyadenylation signal is underlined with a wavy line.

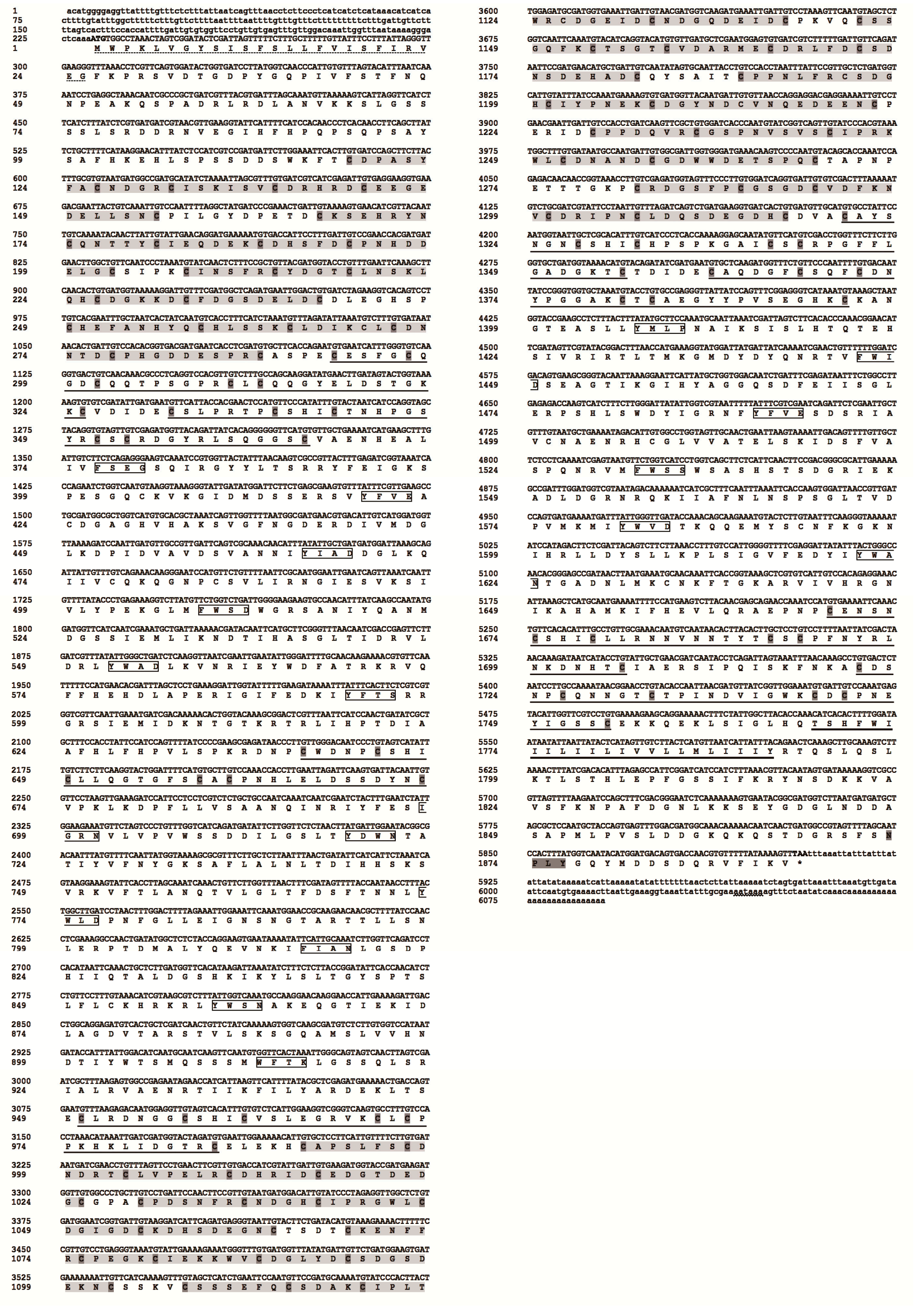

Figure S2. Nucleotide and amino acid sequence of vitellogenin receptor (*VgR*) from *Panonychus citri* (*PcVgR*). The ATG start codon and TAA stop codon are in bold, and the signal peptide is underlined with a dashed line. The Cysteine residues are represented by the letter C in a gray background. The two clusters of ligand binding domains (LBDs) are shown in gray shading, and the epidermal growth factor (EGF)-precursor domains are underlined. YWXD repeats are indicated by open boxes. The putative transmembrane domain is identified by a bold underline, and the NPLY internalization signals are shown using dark-shaded frames. The polyadenylation signal is underlined with a wavy line.
